# Frailty and post-operative delirium influence on functional status in patients with hip fracture: the GIOG 2.0 study

**DOI:** 10.1007/s40520-023-02522-8

**Published:** 2023-08-05

**Authors:** Chiara Maria Gandossi, Antonella Zambon, Maria Cristina Ferrara, Elena Tassistro, Giuseppe Castoldi, Francesca Colombo, Chiara Mussi, Emilio Martini, Giuseppe Sergi, Alessandra Coin, Giovanni Zatti, Caterina Trevisan, Stefano Volpato, Andrea Ungar, Giuseppe Bellelli, GIOG 2.0 Study Group, Società Italiana di Gerontologia e Geriatria (SIGG), Maria Lia Lunardelli, Enrico Benvenuti, Stefania Maggi, Alberto Pilotto, Antonella Barone, Amedeo Zurlo, Monica Pizzonia, Raffaele Antonelli Incalzi, Luigi Residori, Paola Cena, Paolo Mazzola, Maurizio Corsi, Alessio Greco, Riccardo Galluccio, Alice Riccò, Luca Molteni, Andrea Poli, Chiara Bendini, Alice Ceccofiglio, Gaia Rubbieri, Giulio Mannarino, Alessandro Cartei, Eleonora Barghini, Ilaria Del Lungo, Silvia Tognelli, Chiara Bandinelli, Giulia Venturelli, Alberto Cella, Chiara Ceolin, Labjona Haxhiaj, Alice Laudisio, Luigi Residori, Martina Bonetto, Maria Grazia Valsecchi

**Affiliations:** 1 School of Medicine and Surgery, Milano-Bicocca University, Milan, Italy; 2 Unit of Biostatistics, Epidemiology and Public Health, Department of Statistics and Quantitative Methods, University of Milano Bicocca, Milan, Italy; 3 Biostatistics Unit, IRCCS Istituto Auxologico Italiano, Milan, Italy; 4 School of Medicine and Surgery, University of Milano-Bicocca, Milan, Italy; 5 Bicocca Center of Bioinformatics, Biostatistics and Bioimaging (B4 Centre), School of Medicine and Surgery, Milano-Bicocca University, Monza, Italy; 6 Orthopedic Unit, Carate Brianza Hospital, ASST Brianza, Vimercate, MB Italy; 7 Orthogeriatric Unit, University of Modena and Reggio Emilia, Modena, Italy; 8 Department of Medicine, Geriatrics Unit, University of Padua, Padua, Italy; 9 Orthopedic Unit, IRCCS S. Gerardo Hospital, Monza, Italy; 10 Department of Medical Sciences, University of Ferrara, Ferrara, Italy; 11 Orthogeriatric Unit, Arcispedale S. Anna, University Hospital of Ferrara, Ferrara, Italy; 12 Geriatrics and Intensive Care Unit, University of Florence and AOU Careggi, Florence, Italy; 13 Orthogeriatric Unit, IRCCS San Gerardo Hospital, Monza, Italy

**Keywords:** Delirium, Frailty, Functional outcome, Hip fracture, Orthogeriatric

## Abstract

**Background:**

This study analyzes the effect of frailty and Post-Operative Delirium (POD) on the functional status at hospital discharge and at 4-month follow-up in patients with hip fracture (HF).

**Methods:**

Multicenter prospective observational study of older patients with HF admitted to 12 Italian Orthogeriatric centers (July 2019-August 2022). POD was assessed using the 4AT. A 26-item Frailty Index (FI) was created using data collected on admission. The outcome measures were Cumulated Ambulation Score (CAS) ≤ 2 at discharge and a telephone-administered CAS ≤ 2 after 4 months. Poisson regression models were used to assess the effect of frailty and POD on outcomes.

**Results:**

984 patients (median age 84 years, IQR = 79–89) were recruited: 480 (48.7%) were frail at admission, 311 (31.6%) developed POD, and 158 (15.6%) had both frailty and POD. In a robust Poisson regression, frailty alone (Relative Risk, RR = 1.56, 95% Confidence Intervals, CI 1.19–2.04, p = 0.001) and its combination with POD (RR = 2.57, 95% CI 2.02–3.26, p < 0.001) were associated with poor functional status at discharge. At 4-month follow-up, the combination of frailty with POD (RR 3.65, 95% CI 1.85–7.2, p < 0.001) increased the risk of poor outcome more than frailty alone (RR 2.38, 95% CI 1.21–4.66, p < 0.001).

**Conclusions:**

POD development exacerbates the negative effect that frailty exerts on functional outcomes in HF patients.

**Supplementary Information:**

The online version contains supplementary material available at 10.1007/s40520-023-02522-8.

## Introduction

Hip fractures (HFs) are common in older people: yearly 1,600,000 HF occur worldwide, of which more than 123,000 in Italy [1]. The consequences are relevant: nearly one-third of patients die within one year after HF, and about half of the survivors do not regain their pre-fracture functional status [2, 3]. These figures threaten the sustainability of national healthcare systems [4] as the population is aging and the number of HF is expected to increase [5].

Frailty is a geriatric syndrome characterized by excessive vulnerability to stressors and impaired ability to maintain individual homeostasis. Frailty is a predisposing factor for postoperative delirium (POD) [6, 7].

Both frailty and POD are associated with an increased risk of negative outcomes, including poor functional status and disability, suggesting that these conditions may concur to affect the patient’s health status after HF [8–11].

However, studies focusing on the combined effect of frailty and POD on the functional status of HF patients after surgical repair are lacking [8, 9, 12–14].

The aim of this study is to explore the effect of frailty, POD, and their combination on the risk of poor functional status at hospital discharge and four months after discharge in an Italian multicenter cohort of patients with HF.

## Methods

### Setting and sample

The GIOG 2.0 is an unfunded, multicenter, prospective, observational study to evaluate the practice of care and key-performance indicators in 12 Italian orthogeriatric centers. The data presented in this study refer to the period between July 1, 2019, and August 31, 2022. Inclusion criteria for the study were: age ≥ 65 years, proximal HF requiring urgent surgical intervention, the willingness of the patient or his/her caregiver (if the patient was unable) to sign an informed consent form, and ability to speak Italian. Exclusion criteria were a diagnosis of distal HF, metastatic cancer, or a life expectancy of less than one month (according to the physician’s judgment). The study was conducted in accordance with the EU Regulation 2016/679 and the EU Directive 2016/680, and the protocol was approved by the Brianza Institutional Review Board. The RedCap Cloud platform was used to ensure data confidentiality, and data were anonymized (https://eulogin.redcapcloud.com/#cid=nph2020&act=list&studyId=343).

### Frailty index

A Frailty Index (FI) of health deficits was operationalized according to a standard procedure [15], that includes the ascertainment of the presence of medical conditions, disabilities, signs, and symptoms based on the information documented in their medical records and reported by their family members with reference to the pre-fracture health status. The values (median and IQR or number and percentage) of the variables used to compute our 26-item FI are reported in Supplementary Table 1. A score of 0 for the absence of deficit and 1 for the presence of deficit was assigned for each variable. The FI score was calculated for each patient by dividing the sum of observed deficits by the sum of all measured variables. For example, if a person had 10/26 altered items, the corresponding FI score was 0.38. As in a previous study, a cut-off ≥ 0.25 was used to define frail patients [16].

### Diagnosis of POD

From the first to the third day after surgery, each patient was evaluated daily for POD occurrence by a geriatrician using the 4AT test [17], a tool with a sensitivity of 88% and a specificity of 88% for the diagnosis of delirium [18]. All patients who scored > 4 at 4AT and showed symptoms of delirium for at least one day after surgery were classified as having POD. On holidays, when assessors were not at the hospital and could not assess 4AT, information on delirium was obtained from a review of daily medical and nursing notes, as in previous studies [14, 19].

### Other measurements

On admission, all patients underwent a Comprehensive Geriatric Assessment (CGA), which included data on demographics (age, sex, and living arrangements), mobility status (Standardised Audit of Hip Fracture in Europe-SAHFE) [20], and cognitive status (Short Portable Mental Status Questionnaire, SPMSQ) [21]. During hospitalization, we also assessed the American Society of Anesthesiologists (ASA) Classification score [22], time between admission and surgery, type of HF, and type of anesthesia.

### Medical care and rehabilitation

At each center, patients were examined daily by an orthopedic surgeon and a geriatrician, and post-operative rehabilitation was provided by a team of physical therapists. Treatment protocols included standing and walking exercises aimed at improving the patient’s functional status.

### Outcome measure

The outcome measure was evaluated with the Cumulated Ambulation Score (CAS) [23] at hospital discharge and at 4 months. We chose a 4-month follow-up period for assessing functional status because previous studies have indicated that most of functional improvement after hip fracture occurs within the first 3 months, with minimal further improvements expected beyond this time frame [24]. CAS is a score that assesses the patient’s independence in three essential functions: transfer in and out of bed, sit to stand from a chair, walking with or without aid. For each function, 2 points are given if the patient can complete the task without help, 1 point if the patient requires help, and 0 points if the patient cannot perform the task. Poor functional status at discharge was defined by a CAS ≤ 2.

The outcome measure at the 4-month follow-up was a telephone-administered CAS in which either the patient or the caregiver was asked to report the patient’s independence in the tasks evaluated by the CAS (i.e., transfer from sitting to supine to sitting, transfer from sitting-to-standing-to-sitting and walking with or without an appropriate aid). We used the same scoring system as the original CAS, and we defined the presence of poor functional status at 4 months with a total score ≤ 2.

### Statistical analyses

Continuous variables are reported as median and interquartile range (IQR) because their distribution was not normal. Qualitative variables are reported as frequencies and percentages. Statistical significance between groups (frails vs non-frails) was evaluated using the Wilcoxon test for continuous variables and the Chi-square test for categorical variables.

To evaluate the association of frailty, POD, and their combination with the outcome measure at hospital discharge, a 4-group variable (frailty alone, POD alone, frailty plus POD, neither) was created and included in a robust Poisson regression [25], adjusting for confounders selected a priori based on their significance in univariate analysis (age, sex, type of fracture, 48-h surgical delay, type of anesthesia). A similar Poisson regression analysis was performed in the cohort of patients who had the 4-month follow-up data, using telephone-administered CAS as the outcome measure. Results were adjusted for the likelihood of patients being lost or dead at follow-up. Association estimates were reported as relative risk (RR) and corresponding 95% confidence intervals (95%CI). All tests were two-sided, and we considered a p value < 0.05 as significant. All analyses were performed using SAS software (version 9.4; SAS Institute).

## Results

**Fig. 1:**
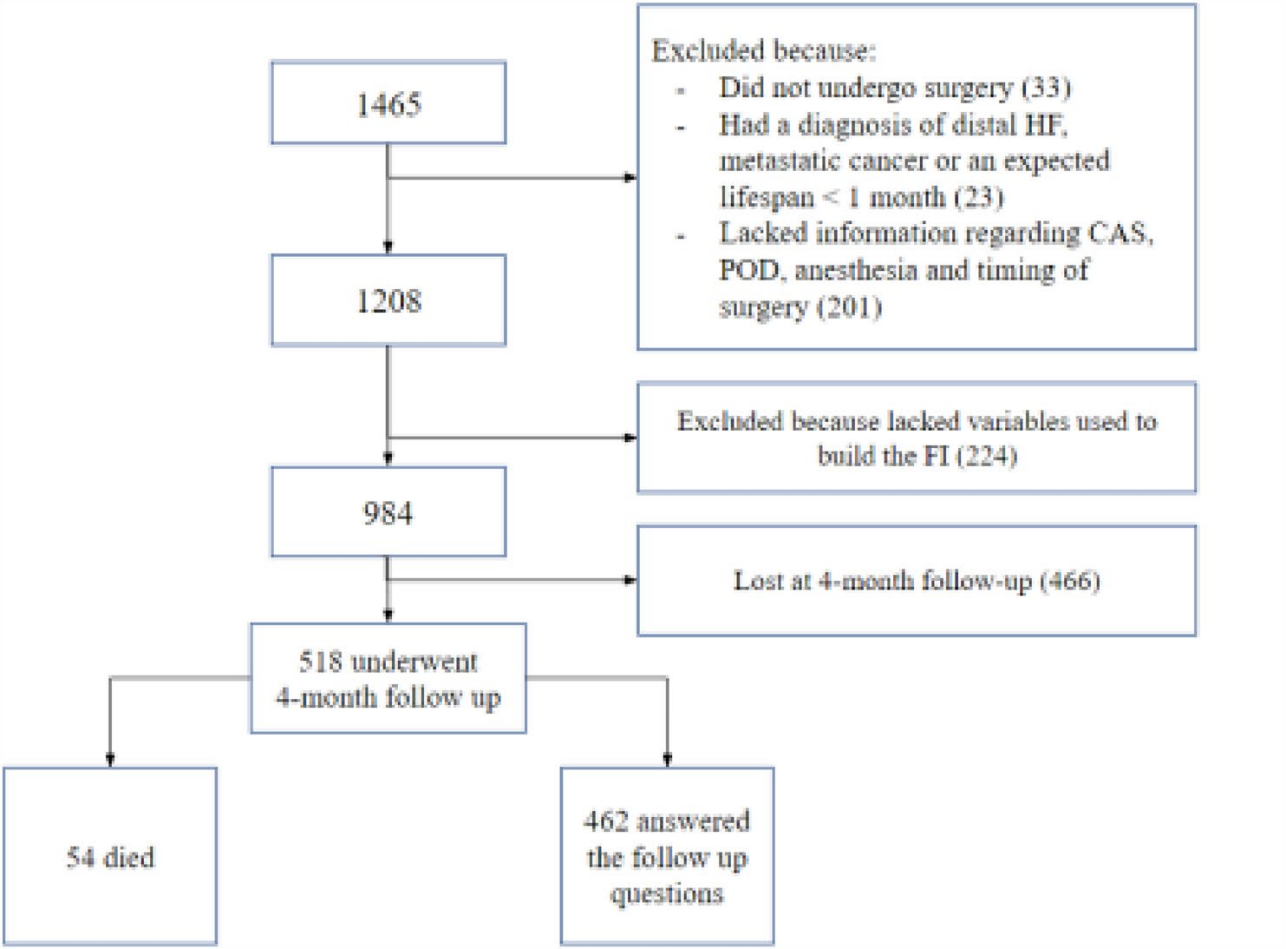
Flow chart of the patients’ selection process

Twelve centers participated in the baseline recruitment. Figure 1 shows the flowchart of the patients in the study. A total of 1465 patients’ records were collected at hospital discharge. Of these, 481 had one or more exclusion criteria, leaving a final population of 984 patients. The 4-month follow-up was obtained in 518 patients, recruited from 8 centers. Of these patients, 54 died, leaving a final sample of 462 patients. Differences between patients who underwent the 4-month follow-up and those who did not are shown in Supplementary Table 2.

Table 1 shows clinical features and outcomes at discharge of patients recruited at baseline, for the entire population and according to the presence of frailty. The median age was 84 (IQR 79–89) years and only 241 participants were males (24.5%). Almost all patients (938, 95.3%) lived at home and only 46 (4.7%) were institutionalized. The median number of drugs taken daily was 4 (IQR 2–6). Before the fracture, more than half of the sample already had walking impairment, and the median SPMSQ score was 3 (IQR 1–6), suggesting mild cognitive impairment. Inter‐trochanteric fractures were more common (45%) than intracapsular fractures (43.6%), which corresponded to a prevalent use of intramedullary nails for osteosynthesis (51.4%). Regional anesthesia was used in 879 (87.6%) patients, and surgical delay (i.e., ≥ 48 h from hospital admission to surgery) occurred in 21.7% of patients. POD developed in 311 (31.6%) patients and the median length of hospital stay was 9 (IQR 7–12) days. At discharge, 31.7% of patients had poor functional status (i.e., CAS score ≤ 2); most of them were discharged to a rehabilitation facility (58%), 33.6% returned home, and 6.3% to a nursing home.

**Table 1:** Clinical features and outcomes of the patients recruited, as a whole sample and according to Frailty Index (FI) score

Variable	Full sample (n = 984)	FI < 0.25 (n = 504)	FI ≥ 0.25 (n = 480)	p value
Collected at hospital admission				
Age, years	84 (79–89)	82 (77–87)	86 (81–90)	< .0001
Male	241 (24.5)	113 (22.4)	128 (26.7)	0.122
Living at home	938 (95.3)	498 (98.8)	440 (91.6)	< .0001
Number of daily drugs	4 (2–6)	3 (1–5)	5 (3–7)	< .0001
Unable to walk	15 (1.6)	1 (0.2)	14 (3)	< .0001
Able to walk only indoor (with aid)	521 (54.4)	152 (31.0)	369 (78.9)	
Able to walk outdoor with or without aid	422 (44.0)	337 (68.8)	85 (18.2)	
SPMSQ score	3 (1–6)	1 (0–3)	5 (2–10)	< .0001
Hemoglobin serum levels (g/dl)	12 (11–13)	12.4 (11.4–13.5)	11.7 (10.5–12.5)	< .0001
Related to intervention				
Fracture type				
Intracapsular	429 (43.6)	241 (47.8)	188 (39.2)	0.015
Inter‐trochanteric	446 (45.3)	207 (41.1)	239 (49.8)	
Other	109 (11.1)	56 (11.1)	53 (11)	
ASA score	3 (2–3)	3 (2–3)	3 (2–3)	< .0001
Regional anesthesia	862 (87.6)	446 (88.5)	416 (86.7)	0.385
Hip arthroplasty	400 (40.6)	221 (43.8)	179 (37.3)	< .0001
Intramedullary nail	506 (51.4)	230 (45.6)	276 (57.5)	
Other	78 (7.9)	53 (10.6)	25 (5.1)	
Surgical delay (≥ 48 h)	214 (21.7)	98 (19.4)	116 (24.2)	0.072
Related to post‐surgical course				
Postoperative delirium	311 (31.6)	83 (16.5)	228 (47.5)	< .0001
Outcomes collected at discharge				
CAS	3 (2–3)	3 (3–4)	3 (1–3)	< .0001
Length of hospital stay, days	9 (7–12)	9 (7–12)	9 (7–13)	0.126
Discharged to home	330 (33.6)	186 (37)	144 (30.1)	< .0001
Discharged to rehabilitation	570 (58)	304 (60.4)	266 (55.5)	
Discharged to nursing home	62 (6.3)	10 (2)	52 (10.9)	
Other discharge	20 (2)	3 (0.6)	17 (3.5)	

Values are reported as median and (Interquartile range) or number (%)

Wilcoxon test for continuous variables and chi-square test for categorical variables were used to compare frail and non-frail patients

*MNA* mini nutritional assessment, *SAHFE* scottish audit hip fracture classification, *ADL* activities of daily living, *NMS* new mobility score, *NEWS* national early warning score, *SPMSQ* short portable mental status questionnaire, *ASA* american society of anesthesiologists, *CAS* cumulated ambulation score, *FI* frailty index

Overall, 421 (42.8%) were non frail and didn’t develop delirium, 252 (25.7%) had frailty alone without delirium, 83 (8.4%) were non frail and didn’t develop delirium and 228 (23.1%) had both frailty and delirium. At discharge, the proportion of patients with CAS score ≤ 2 was higher in frail patients (43.3% vs 20.6%), whereas there were no significant differences in the discharge setting.

Clinical characteristics and outcomes of patients who underwent the4-month follow-up are shown in Table 2. Overall, 54 (10.4%) patients died, and 71 (15.4%) of the survivors had poor functional status. Most of the patients were living at home (71.5%), 12.8% were in a rehabilitation ward, and 8.8% were in a nursing home. The mortality of frail patients was threefold higher than in non-frail patients (16.7% vs 4.8% p < 0.001). The percentage of those with poor functional status was 27.2% in frail and 5.9% in non-frail patients. There was a higher proportion of frail patients living in nursing homes compared to non-frail patients.

**Table 2:** Outcomes of the patients who underwent the 4-month follow-up, as a whole sample and according to Frailty Index (FI) score

Variable	Full sample (n = 518)	FI < 0.25 (n = 272)	FI ≥ 0.25 (n = 246)	p value
Mortality	54 (10.4)	13 (4.8)	41 (16.7)	< .0001
Telephone-administered CAS ≤ 2 (N = 462)	71 (15.4)	15 (5.9)	56 (27.2)	< .0001
Residence status (N = 462)				
Home	291 (71.5)	164 (81.2)	127 (61.9)	< .0001
Nursing home	36 (8.8)	7 (3.5)	29 (14.1)	
Rehabilitation	52 (12.8)	26 (12.9)	26 (12.7)	
Other	28 (6.9)	5 (2.5)	23 (11.2)	

Values are reported as median and (Interquartile range) or number (%)

Wilcoxon test for continuous variables and chi-square test for categorical variables were used to compare frail and non-frail patients

*CAS* cumulated ambulation score, *FI* frailty index

Table 3 shows the results of two Poisson regression models to estimate the risk of poor functional status at discharge (panel A) and after 4 months (panel B) according to the presence of frailty and POD, alone or in combination. Frailty alone (RR = 1.56, 95% CI 1.19–2.04, p = 0.01) and frailty plus POD (RR = 2.57, 95% CI 2.21–3.26, p < 0.001) were significantly associated with poor functional status at discharge. At the 4-month follow-up, the interaction between frailty and POD (RR 3.65, 95% CI 1.85—7.2, p < 0.001) increased the risk of negative outcomes more than frailty alone (RR = 2.38, 95% CI 1.21–4.66, p = 0.01.

**Table 3:** Robust Poisson regression models of the variables associated with poor functional status at discharge (CAS ≤ 2) and at 4-month follow up (telephone administered CAS ≤ 2)

Variable	Panel A		Panel B	
	At discharge (N = 984)		At 4-month follow-up (N = 462)	
	RR (95% CI)	p value	RR (95% CI)	p value
Frailty index and post‐operative delirium				
Frailty no/delirium no	1		1	
Frailty yes/delirium no	1.56 (1.19–2.04)	0.0012	2.38 (1.21–4.66)	0.0116
Frailty no/delirium yes	1.37 (0.92–2.02)	0.1197	0.22 (0.03–1.56)	0.1307
Frailty yes/delirium yes	2.57 (2.02–3.26)	< .0001	3.65 (1.85–7.2)	0.0002
Socio‐demographic variables				
Age		0.0059		0.0039
Female sex		0.9465		0.4702
Fracture and intervention covariates				
Inter‐trochanteric/subtrochanteric fracture		0.2304		0.5928
Other types of fracture		< .0001		0.4014
48‐h delay in intervention		0.0946		0.5399
General anesthesia/Sedation		0.1821		0.2001

*RR* relative risk, *95% CI* confidence intervals, *CAS* cumulated ambulation score

## Discussion

This large, multicenter, prospective study of patients with HF recruited in Italian orthogeriatric centers, shows that frailty alone and its combination with POD significantly affect functional status at discharge. Furthermore, the development of POD exacerbates the negative effect that frailty exerts on patient function at 4-month follow-up.

Two recent systematic reviews that included older patients undergoing HF surgery, predominantly examined the association of frailty with mortality, length of hospital stay, risk of complications after surgery, and risk of institutionalization, whereas functional status was relatively understudied [26, 27].

Using a modified 19-item FI, Inoue et al. found that frailty was independently associated with an increased likelihood of poor functional recovery at discharge [15]. In another study, Low et al. found that frailty (measured by the Clinical Frailty Scale) was the strongest independent predictor of poor Functional Independence Measure (FIM) efficiency at discharge, inability to regain pre-fracture mobility, and return home [28]. Furthermore, in a multicenter study of 36,192 patients, frailty, measured by the Hospital Frailty Risk Score (HFRS) based on ICD-10 reports, was associated with a higher risk of poor Barthel Index score at hospital discharge [29]. However, none of these studies examined the patient’s functional status after discharge.

The relationship between POD and functional outcome is supported by a large body of evidence. Ouellet et al. [30] showed that patients who developed POD after HF surgery had a higher risk of poor Barthel Index at discharge. Shi et al. [31] found that patients who developed POD experienced a greater decline in ADL score at 24 and 36 months after surgery compared to their counterparts. The negative effect of POD on functional status was also found in studies that included patients discharged to rehabilitation and long-term care facilities after HF surgery [32–34]. However, none of these studies examined the combined effect of frailty and delirium on subsequent functional status. This is an interesting issue because frailty may predispose to delirium and should act as a confounding variable when examining the association between delirium and functional outcomes.

To our knowledge, only one previous study [12] has examined the effect of frailty and POD on patients’ functional status at hospital discharge. In a cohort of 988 patients undergoing HF surgery, the authors found that frailty, POD, and their combination were independently associated with low CAS scores at discharge. However, this study was based on data from a single center and did not collect information on patients’ status after hospital discharge.

Our work contributes to the evidence in this field by demonstrating that the effect of frailty on functional status extends beyond hospital discharge and that POD interacts with frailty to increase the risk of poor functional outcome 4 months after hospital discharge.

Overall, these data suggest at least three possible interpretations. The most suitable is that POD superimposed on frailty may trigger a downward spiral leading to a negative chain of reactions (i.e., neuroinflammation, brain metabolic insufficiency, neurotransmitters’ imbalance, and others) that self-maintain after discharge and threaten the patient’s recovery [35].

However, it could also be hypothesized that POD hampers functional recovery through various mechanisms, such as a delayed onset of the rehabilitation process and reduced patient engagement. Additionally, POD might represent a marker of poor resilience, suggesting that it could be used as a condition to stratify risk in frail patients [36]. The lack of effect of POD alone on the outcomes at discharge and at 4-month follow-up might be due to the scarcity of patients without frailty who developed POD. The difference between this study and our previous study [12] regarding the effect of POD on functional status at discharge after HF surgery may be explained by the different methods used to assess POD and by the multicenter design of the present study.

Previous systematic reviews have shown that multicomponent non-pharmacological interventions can prevent delirium, decreasing its incidence by more than 40% [37]. Since functional improvement in HF patients occurs mainly within 3 months after HF, the results of the present study suggest that it is important to screen for frailty on hospital admission and to prevent and treat POD not only to improve functional status at discharge but also to reduce the risk of further decline in the longer term [24].

The strengths of this study are the large sample size, the prospective and multicenter design, the use of a standardized approach to evaluate the patient’s clinical status, and the method used to assess POD. Indeed, all patients were assessed using the 4AT, which has shown, good overall performance in diagnostic accuracy for delirium detection in a recent systematic review and meta-analysis [18].

A limitation of the study is that we lost a relevant proportion of patients at the 4-month assessment, which may bias our results at follow-up. However, it may be considered that the multivariate Poisson regression model used to determine the variables associated with poor functional status at 4 months was adjusted for the patient’s likelihood of being lost or dead at follow-up. A second limitation is that functional status at 4 months was determined with a surrogate CAS assessed by telephone interviews because of limited resources (the GIOG 2.0. is not supported by any funding source). Third, the number of items used to compute the FI was lower than that suggested by Searle et al. [38]. However, a growing number of studies have recently been published using FIs that include 20–25 variables and show a good ability to predict negative outcomes in different patient cohorts [39, 40]. Lastly, we must acknowledge the potential for bias in our study results due to the impact of post-hospitalization development of COVID-19 on the extent of functional recovery in certain patients.

## Conclusions

This study shows that in older patients undergoing HF surgery, frailty alone and its combination with POD are significantly associated with poor functional status at discharge. Furthermore, the development of POD exacerbates the negative effect that frailty exerts on functional outcomes after 4 months. The results of the present study highlight the importance of screening for frailty and of preventing and treating POD in patients undergoing HF surgery, in order to improve their functional status at discharge and reduce the risk of medium- and long-term disability.

### Supplementary Information

Below is the link to the electronic supplementary material.

Supplementary file1 (DOCX 23 KB)

## Data Availability

The datasets generated during and/or analysed during the current study are available from the corresponding author on reasonable request.
