# Supplementary material for: Frailty and post-operative delirium influence on functional status in patients with hip fracture: the GIOG 2.0 study
Source: Aging Clin Exp Res. 2023 Aug 5;35(11):2499–506. doi: 10.1007/s40520-023-02522-8 (PMC10628048; doi:10.1007/s40520-023-02522-8)
Supplement: Supplementary file 1 — Supplementary file1 (DOCX 23 KB) [file 40520_2023_2522_MOESM1_ESM.docx]

**Supplementary Table 1.**

| **Variable** | **Full sample**  **(n = 1012)** | **Coding** |
| --- | --- | --- |
| Myocardial infarction, n (%) | 166 (16.4) | No=0, Yes=1 |
| Congestive heart failure, n (%) | 124 (12.25) | No=0, Yes=1 |
| Severe liver disease, n (%) | 7 (0.69) | No=0, Yes=1 |
| Kidney failure, n (%) | 142 (14.03) | No=0, Yes=1 |
| Cancer, n (%) | 89 (8.79) | No=0, Yes=1 |
| Peripheral arterial disease, n (%) | 159 (15.71) | No=0, Yes=1 |
| Cerebral vascular disease, n (%) | 216 (21.34) | No=0, Yes=1 |
| Stroke, n (%) | 23 (2.27) | No=0, Yes=1 |
| Dementia, n (%) | 329 (32.51) | No=0, Yes=1 |
| AIDS, n (%) | 1 (0.1) | No=0, Yes=1 |
| Diabetes, n (%) | 221 (21.84) | No=0, Yes=1 |
| Chronic lung disease, n (%) | 95 (9.39) | No=0, Yes=1 |
| Gastric ulcer, n (%) | 27 (2.67) | No=0, Yes=1 |
| Connective tissue disease, n (%) | 33 (3.26) | No=0, Yes=1 |
| MNA score, median (IQR) | 11 (9-12) | MNA≥8=0, MNA<8=1 |
| Need help with bathing | 289 (29.4) | No=0, Yes=1 |
| Need help with dressing | 273 (27.7) | No=0, Yes=1 |
| Need help with toileting | 370 (37.6) | No=0, Yes=1 |
| Need help with transferring | 346 (35.2) | No=0, Yes=1 |
| Need help with feeding | 541 (55) | No=0, Yes=1 |
| Incontinence | 68 (6.9) | No=0, Yes=1 |
| Unable to get about the house | 493 (50.1) | No=0, Yes=1 |
| Unable to get out of the house | 657 (66.8) | No=0, Yes=1 |
| Unable to go shopping | 701 (71.2) | No=0, Yes=1 |
| Polypharmacy | 450 (45.7) | No=0, Yes=1 |
| Hemoglobin serum levels, (g/dl), median (IQR) | 12 (11-13.5) | Hb>12=0, 10<Hb≥12=0.5, Hb≤10=1 |

Note: Values are reported as median and (Interquartile range) or number (%)

Abbreviations: AIDS, Acquired Immune Deficiency Syndrome; MNA, Mini Nutritional Assessment; Hb, Hemoglobin serum levels.

**Supplementary Table 2.**

| **Variable** | **Patients with 4-month follow-up(n=462)** | **Patients without 4-month follow-up(n=462)** | **p value** |
| --- | --- | --- | --- |
| *Collected at hospital admission* | | | |
| Age, years | 83 (79-88) | 85 (80-89) | 0.0026 |
| Male | 122 (26.4) | 119 (22.8) | 0.1888 |
| Residence at hospital admission | | | |
| At home | 446 (96.6) | 492 (94.3) | 0.0767 |
| Nursing home | 16 (3.5) | 30 (5.7) |  |
| Number of daily drugs | 4 (1-6) | 4 (3-6) | 0.0003 |
| Unable to walk | 5 (1.1) | 10 (1.9) | 0.0087 |
| Able to walk only indoor (with aid) | 226 (50.9) | 295 (57.4) |  |
| Able to walk outdoor with or without aid | 213 (48) | 177 (40.6) |  |
| SPMSQ score | 2 (1-5) | 3 (1-7) | 0.0025 |
| Hemoglobin serum levels (g/dl) | 12.2 (11.1-13.3) | 12 (10.9-12.9) | <.0001 |
| *Related to intervention* | | | |
| Fracture type | | | |
| Intracapsular | 200 (43.3) | 229 (43.9) | 0.3635 |
| Inter‐trochanteric | 204 (44.2) | 242 (46.4) |  |
| Other | 58 (12.5) | 51 (9.8) |  |
| ASA score | 3 (2-3) | 3 (2-3) | 0.0003 |
| Regional anesthesia | 372 (80.5) | 490 (93.9) | <.0001 |
| Type of surgery | | | |
| Hip arthroplasty | 180 (39) | 220 (42.1) | 0.62932 |
| Intramedullary nail | 238 (51.5) | 268 (51.3) |  |
| Other | 44 (9.5) | 34 (6.4) |  |
| Surgical delay (≥48 h) | 101 (21.9) | 113 (21.6) | 0.9353 |
| *Related to post‐surgical course* | | | |
| Postoperative delirium | 146 (31.6) | 165 (31.6) | 0.998 |
| *Outcomes collected at discharge* | | | |
| CAS, median (IQR) | 3 (2-3) | 3 (2-3) | 0.1135 |
| Length of hospital stay, days | 9 (7-11) | 10 (8-13) | <.0001 |
| Discharge destination | | | |
| Home | 153 (33.1) | 177 (34) | 0.0004 |
| Rehabilitation | 281 (60.8) | 289 (55.6) |  |
| Nursing home | 19 (4.1) | 43 (8.3) |  |
| Other | 9 (1.9) | 11 (2.1) |  |

Note: Values are reported as median and (Interquartile range) or number (%)

Wilcoxon test for continuous variables and chi-square test for categorical variables were used to compare frail and non-frail patients.

Abbreviations: MNA, Mini Nutritional Assessment; SAHFE, Scottish Audit Hip Fracture Classification; ADL, Activities of Daily Living; NMS, New Mobility Score; NEWS, National Early Warning Score; SPMSQ, Short Portable Mental Status Questionnaire; ASA, American Society of Anesthesiologists; CAS, Cumulated Ambulation Score; FI, Frailty Index.
